# A Simple and Rapid LC-MS/MS Method for the Quantification of Nirmatrelvir/Ritonavir in Plasma of Patients with COVID-19

**DOI:** 10.1155/2024/6139928

**Published:** 2024-03-06

**Authors:** Xiujing Zhu, Lin Li, Bing Dai, Zhijun Liu, Zhipeng Wang, Lili Cui, Shouhong Gao, Wansheng Chen, Xia Tao, Deduo Xu

**Affiliations:** ^1^Department of Pharmacy, Second Affiliated Hospital of Naval Medical University, Shanghai 200003, China; ^2^College of Traditional Chinese Medicine, Yunnan University of Traditional Chinese Medicine, Kunming, Yunnan 650500, China; ^3^Department of Nephrology, Second Affiliated Hospital of Naval Medical University, Shanghai 200003, China

## Abstract

The combined prescriptions of nirmatrelvir/ritonavir and other drugs are limited due to potential drug-drug interactions, so therapeutic drug monitoring (TDM) becomes particularly important. In this study, a liquid chromatography-tandem mass spectrometry (LC-MS/MS) method was established for determination of the nirmatrelvir/ritonavir in plasma of patients with COVID-19, providing technical and theoretical support for the TDM. Plasma samples were processed by protein precipitation using acetonitrile, and analytes were separated on an Agilent Poroshell 120 SB-C18 (2.1 × 75 mm, 2.7 *μ*m) column at 35°C. Acetonitrile and 0.1% formic acid in water (52 : 48) were utilized as the mobile phases at a flow rate of 0.3 mL/min. In the multiple reaction monitoring (MRM) mode, nirmatrelvir and ritonavir were monitored using precursor/product ions: *m*/*z* 500.2/110.1 and 721.3/296.1, respectively, with selinexor as the internal standard. The linear range of both analytes was 2.0 ng/mL to 5000 ng/mL with good inter- and intraday precision and accuracy, and the recovery was 92.0%–107% for nirmatrelvir and 85.7%–106% for ritonavir. Finally, this method was successfully applied to monitor the exposure levels of nirmatrelvir/ritonavir in plasma samples from hemodialysis patients.

## 1. Introduction

Coronavirus disease 2019 (COVID-19) is a serious infectious disease caused by severe acute respiratory syndrome coronavirus type 2 (SARS-CoV-2), which has caused a large-scale epidemic worldwide. Multiple generations of variants have occurred during the epidemic, and the fifth generation variant, the Omicron variant, is currently the most common [1]. It has been reported that although the viral genome of SARS-CoV-2 is frequently mutated, nirmatrelvir/ritonavir shows an effective antiviral effect against recent coronavirus mutants [2]. The oral form nirmatrelvir/ritonavir was an effective new drug against COVID-19, compared with remdesivir and molnupiravir [3, 4]. The results of clinical studies conducted on COVID-19 patients with underlying disease factors showed that the risk of hospitalization or death caused by administration of nirmatrelvir/ritonavir was reduced by 89% within 3 d of symptom onset and by 88% within 5 d [5–7].

Ritonavir has been used as a pharmacokinetic enhancer for HIV protease inhibitors, such as darunavir and lopinavir, which are metabolized by cytochrome P450 3A4 (CYP3A4) [7]. Nirmatrelvir is a peptidomimetic inhibitor of SARS-CoV-2 main protease (also referred to as 3C-like protease, 3CLpro). It can prevent virus replication by inhibiting SARS-CoV-2 main protease to make it unable to process multiprotein precursors [8]. CYP3A4 plays an important role in its metabolism, indicating that the combination with ritonavir, a potent CYP3A4 inhibitor, can increase the serum concentration of nirmatrelvir, thereby increasing the therapeutic concentration [5].

However, its potential for enzyme-inhibiting drug-drug interactions should be carefully considered in combination with other prescribed medications. Among them, CYP3A is mainly involved in the metabolism of most immunosuppressive drugs (ISDs), with narrow treatment scopes [9]. This makes it particularly challenging to administrate the combinations of nirmatrelvir/ritonavir and ISDs. As there is effective inhibition of CYP3A by ritonavir, the exposure of ISD will be significantly increased, which will increase the side effects of the drug. Although toxicity can be prevented by reducing the dose of ISDs, the treatment should be prudent if close monitoring, including TDM, is not feasible [10, 11]. Some recommendations were made by the investigators that tacrolimus should be discontinued or that minimal doses should be administered on the first day and that cyclosporine should be reduced by 80% during antiviral therapy [12].

Hiremath et al. [13] reported that patients with low immune function had higher morbidity and mortality of COVID-19, for example, the patients with advanced chronic kidney disease (CKD) and patients with kidney failure. A study summarized the trial registry which reported that 45% of COVID-19 trials excluded CKD patients [14]. Although some studies have proved the applicability of nirmatrelvir/ritonavir in COVID-19 patients in low dose [15, 16], it is still necessary to pay close attention to the blood concentration of CKD patients. At present, the quantitative methods for ritonavir in human plasma are relatively common, but there are few methods for quantifying nirmatrelvir/ritonavir. In this regard, the latest research is that Martens-Lobenhoffer et al. [17] developed and validated a LC-MS/MS method to simultaneously quantify the content of nirmatrelvir/ritonavir in patients with COVID-19. However, the running time of this method was long, and the total cycle time including balancing was 13 min, which hindered the detection of large batch samples. Before this, there were literature reports [18, 19] about quantifying nirmatrelvir/ritonavir in biological matrix by LC-MS/MS, but these methods were usually narrow in the linear range, which was 10.0 ng/mL to 1000 ng/mL for ritonavir, 40.0 ng/mL to 4000 ng/mL, or 50.0 ng/mL to 500.0 ng/mL for nirmatrelvir.

To better carry out the TDM of nirmatrelvir/ritonavir and ensure the safety and efficacy of medication, a rapid, reliable method with sufficient linear range is urgently needed to simultaneously quantify nirmatrelvir and ritonavir in patients' plasma. Thus, this study presented the development and validation of a fast, sensitive, and reliable LC-MS/MS method with 3.65 min run time for the quantification of nirmatrelvir/ritonavir in human plasma.

## 2. Materials and Methods

### 2.1. Chemicals and Reagents

Nirmatrelvir (purity > 98%, lot: N17HS201609) and ritonavir (purity > 99%, lot: H03D9Z76283) were purchased from Shanghai Sunny Biotech Co., Ltd (Shanghai, China). Selinexor (internal standard, lot: C13J11L117641, purity > 99%) was supplied by Shanghai Yuanye Biotechnology Co., Ltd. (Shanghai, China). HPLC-grade methanol (MeOH) and acetonitrile (ACN) were obtained from Merck (Merck Company, Darmstadt, Germany). HPLC-grade formic acid was from McLean Bio-Tech Co., Ltd (McLean, Shanghai, China). Distilled water was purchased from Shenzhen Watsons Distilled Water Co., Ltd (Shenzhen, China).

### 2.2. LC/MS-MS Instrumentation

The experiments, which included method development and application, were carried out on the system of Agilent 1290 UHPLC in series with Agilent 6460A mass spectrometer, and the system also included online degasser, binary pump, automatic sampler, and column oven. Agilent MassHunter data processing software (version 6.00) was used to collect and analyze the data.

### 2.3. Liquid Chromatographic Conditions

The column in this experiment was Agilent Poroshell 120 SB-C18 (2.1 × 75 mm, 2.7 *μ*m, Agilent, USA) with the temperature kept at 35°C. The mobile phase was made up of 48% A phase (water with 0.2% formic acid) and 52% B phase (ACN) in isocratic elution program because the symmetrical peaks and appropriate retention times were obtained for two analytes. The total running time was 3.65 min. The flow rate was 0.3 mL/min.

### 2.4. Mass Spectrometry Conditions

**Figure 1:**
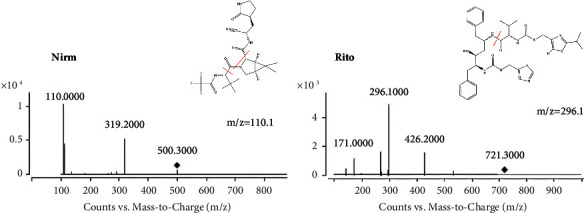
Production mode showing the production and fragmentary structure of nirmatrelvir and ritonavir.

The analytes were ionized in a positive ionization mode using an ESI source with a capillary voltage of 4500 V. In the MRM mode, all data gathering was done, and Figure 1 showed the product ions and fragmentary structures of nirmatrelvir and ritonavir. The drying gas, sheath gas, and sprayer gas are all nitrogen. The nebulizer pressure was 310.275 kPa (45 psi). The drying gas was moved at a rate of 10 L/min while being heated to 320°C. The sheath gas's temperature was fixed at 300°C, and the flow rate was 12 L/min. The collision gas was a high-quality nitrogen gas, set at 0.2 MPa. The mass spectrometry parameters for nirmatrelvir, ritonavir, and IS are presented in Table 1.

### 2.5. Preparation of Standard Solution and Quality Control Sample

The stock solutions with concentrations of 1000 *μ*g/mL, 100.0 *μ*g/mL, and 10.0 *μ*g/mL were obtained by accurately weighing 2.0 mg of nirmatrelvir and ritonavir, respectively, and dissolved in MeOH. These stock solutions were transferred into 1.5 mL tubes and frozen at −80°C. The working solutions were prepared by diluting these stock solutions with MeOH, and then working solutions were combined and diluted with blank plasma to obtain calibration standards at the following concentrations: 2.0, 5.0, 10.0, 20.0, 50.0, 100.0, 200.0, 500.0, 1000, 2000, and 5000 ng/mL. Quality control (QC) samples of both analytes were also prepared in the same way separately, with concentrations of 5.0, 1000, and 2000 ng/mL (low-middle-high). At −80°C, 1.0 mg/mL of IS stock solution was stored and was diluted freshly with MeOH to obtain 20.0 ng/mL IS solution when needed.

### 2.6. Sample Pretreatment

A simple protein precipitation method was used for the pretreatment of samples. A 1.5 mL Eppendorf tube was filled with 100 *μ*L of the plasma sample and 200 *μ*L of ACN (containing 20 ng/mL IS), and then the mixture was vortexed for 2 min. After centrifugation at 14 300 × g for 10 min, 100 *μ*L of the supernatant was diluted with 100 *μ*L of the initial mobile phase, and then we transferred the diluted solution to HPLC vials with glass inserts, and 5 *μ*L was injected into the system.

### 2.7. Methodological Study and Data Analysis

The developed method was validated according to the current bioanalytical guidelines from the U.S. Food and Drug Administration (FDA) and China Pharmacopoeia (version 2020). The following items were validated: specificity, linearity, interday and intraday precision and accuracy, linearity, matrix effect and recovery, carry-over, dilution effect, and stability [20, 21].

Specificity was verified using blank matrix, IS spiked, LLOQ, and real samples from 6 different lots.

The linearity of the analytes in three lots was assessed on different days (at least two days), and at least three calibration curves were evaluated in each lot. The low point of standard curve was the LLOQ.

Five QC in three concentration levels and LLOQ samples were prepared in parallel and measured on different days (at least two days) to obtain the interday and intraday precision and accuracy. The precision shows the stability of the instrument, and the accuracy proves the stability of analytes which was calculated using the concentrations of analytes.

The recovery was the percentage of the peak area of spiked samples versus spiked postextraction samples in the same concentration, and the matrix effect was defined as the ratio of the peak area in spiked postextraction samples to the peak area in solvent-substituted samples in the same concentration.

The carry-over was evaluated by injecting the blank sample, and after injection, the highest concentration of the calibration standard was measured.

The dilution effect was assessed by diluting the samples with a concentration higher than the upper limit of quantitation (ULOQ) into the calibration range and comparing the measured concentration with the nominal concentration.

The stability, including room temperature stability (4 h), standing stability (0 h, 12 h, 24 h), three freeze-thaw cycles, and long-term stability (1 month at −80°C), was evaluated using QC samples at three concentration levels.

### 2.8. Patient Enrollment and Sample Collection

The research protocol was reviewed and approved by the Ethics Committee of Shanghai Changzheng Hospital, and informed consent was signed by every patient. The hemodialysis patients infected with SARS-CoV-2 were enrolled and treated with nirmatrelvir/ritonavir. The treatment regimen was nirmatrelvir/ritonavir, 150 mg/100 mg, q12 h, D1; nirmatrelvir/ritonavir, 150 mg/100 mg, qd, D2-D5. Blood samples were collected at trough point and 3 h and 12 h after drug administration. To assess the influence of dialysis to the drug exposure, this study further collected the sample in the dialysis procedure. Each blood sample was collected in the EDTA-3K tube, and the plasma was transferred to cryopreservation in −80°C after centrifuge at 4500 × g, room temperature for 10 min. Sample preparation and processing were accomplished within 1 h.

## 3. Results and Discussion

### 3.1. LC/MS-MS Optimization

In this experiment, the effects of nirmatrelvir/ritonavir in the positive ionization mode and negative ionization mode were compared to find a more suitable detection method, and it was concluded that the response value of the positive ionization mode was higher than that of the negative ionization mode. In the selection of chromatographic columns, different columns (ZORBAX SB-C8, Agilent Poroshell 120 SB-C18, Atlantis T3-C18) were tested. Nirmatrelvir and ritonavir were found to have better peak shapes, responses, and retention times on an Agilent Poroshell 120 SB18 column. The additive ammonium acetate was found to suppress ionization, while formic acid provided higher ionization and therefore promoted higher desirable responses.

### 3.2. Sample Extraction

Plasma samples were preprocessed using protein precipitator methanol and acetonitrile and so on in different ratios (1 : 2, 1 : 3). The acetonitrile in 1 : 2 ratio resulted in higher extraction recovery (70%–80%) although the matrix effect was slightly less steady than 1 : 3 ratio (RSD % less than 7% vs. RSD % less than <10%). Besides, the retention time was increased by 1 min too compared with other precipitators. The supernatant after protein precipitation was further diluted with diluents MeOH, ACN, mobile phase, and so on in different volume ratios. Dilution of the supernatant with an equal volume of the initial mobile phase showed highest improvements in responses (90%–100%). Other pretreatment methods were not evaluated as satisfactory results for recovery and matrix effects had been obtained. The final sample pretreatment method was determined as 1 : 2 protein precipitation using two-fold volume of ACN, and the supernatant was diluted with initial mobile (1 : 1).

### 3.3. Method Validation

#### 3.3.1. Specificity

**Figure 2:**
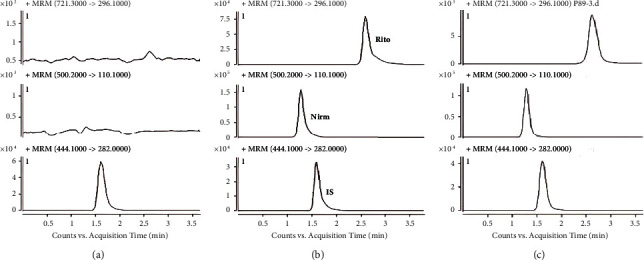
Representative MRM chromatograms for the specificity of analytes. (a) Blank sample spiked with IS; (b) blank sample spiked with LLOQ nirmatrelvir/ritonavir and IS; (c) real sample IS.

Blank plasma matrices from six different individuals were selected to examine the specificity of the method. Finally, the retention times of nirmatrelvir, ritonavir, and the IS were 1.28 min, 2.62 min, and 1.61 min, respectively. The results showed that the endogenous components interfering with the analytes and IS were not presented in the six different blank plasma matrices, indicating good specificity of the method. The typical chromatogram is shown in Figure 2.

#### 3.3.2. Linearity of Calibration Curves and Lower Limited of Quantification (LLOQ)

The calibration curves of both analytes were regressed using the ratio of peak areas of analyte/IS versus the nominal concentration under the 1/*x*^2^ weighing factor, and the equation of the calibration curve of nirmatrelvir was *y* = 37.5581 *∗x* + 8.6239 (*r* = 0.9985) and that of ritonavir was *y* = 30.3743 *∗* *x* − 0.0019 (*r* = 0.9956). The results showed that the linear relationship of analytes was good in the range of 2.0–5000 ng/mL, and the LLOQs of the method were verified to be 2.0 ng/mL. The deviations of back-calculation of every calibration standard were within ±15%. The regression parameters of the calibration curves are shown in Table 2.

#### 3.3.3. Inter- and Intraday Precision and Accuracy

The inter and intraday accuracy and precision were investigated in three batches in three concentration levels along with the linearity assessment. The data showed that the inter- and intra-accuracy (RE %) of nirmatrelvir and ritonavir was within ±15%, and inter- and intraprecision (RSD %) was less than 15%. The data met the requirements of pharmacopoeia, as shown in Table 3.

#### 3.3.4. Recovery and Matrix Effect

The extraction recovery and matrix effect were performed using QC samples at low, middle, and high concentrations of nirmatrelvir and ritonavir (5.0, 1000, and 2000 ng/mL), in three replicates. It was concluded that the recovery and matrix effect of nirmatrelvir were in the range of 92.0%–107%, 87.1%–97.8% and those of ritonavir were 85.7%–106%, 87.8%–112%. The RSD % of recovery and matrix effect was 1.86%–6.59%, 1.58%–4.23% and that of ritonavir was 7.51%–8.59%, 6.49%–9.90% which were less than 15% and proved that the pretreatment method was feasible. The relevant data are presented in Table 4.

#### 3.3.5. Stability

The QC samples with low, middle, and high concentrations (5.0 ng/mL, 1000 ng/mL, 2000 ng/mL) were analyzed immediately after pretreatment or storage under the given conditions to investigate their stability. The results showed that after freeze-thaw cycle treatment for three times, stored at room temperature for 4 h, placed in the automatic sampler for 0 h, 12 h, 24 h after pretreatment, stored at −80°C for 90 d, the RE % and RSD % were within the specified range, proving an acceptable stability. The stability data are shown in Tables 5 and 6.

#### 3.3.6. Dilution Effect

A spiked plasma sample of 10000 ng/mL was prepared from the stock solutions and then diluted 20-fold with blank plasma. After repeating five times, the results showed that the precision (RSD%, 4.42% for nirmatrelvir and 4.80% for ritonavir) and accuracy (RE, −4.11% for nirmatrelvir and 2.13% for ritonavir) met the standards of the Pharmacopoeia.

#### 3.3.7. Carry Over

Carry over was evaluated by measuring the peak area of the blank sample after the ULOQ of the calibration curve for three cycles. The results showed that the peak areas of analytes in the blank sample after detecting the ULOQ sample of nirmatrelvir/ritonavir were less than to 20% of the peak areas of analytes in LLOQ (2.0 ng/mL), which met the requirements.

### 3.4. Method Application

**Figure 3:**
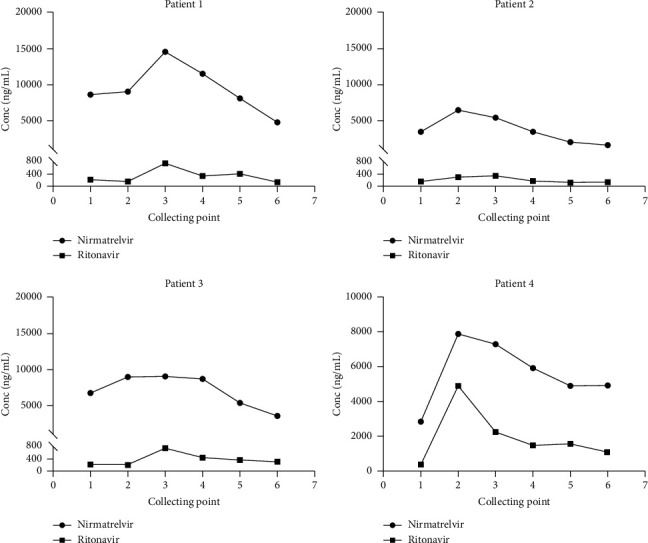
Dynamic variations of nirmatrelvir and ritonavir exposure levels in hemodialysis patients. The collecting points 1, 2, and 3 were the trough, 3 h and 12 h after administration, and the collecting points 4, 5, and 6 were time 0 h, 2 h, and 4 h in the dialysis process.

Totally four hemodialysis patients were enrolled in this study, and all the patients were treated with nirmatrelvir/ritonavir in the same prescription, and the six plasma samples were collected for every patient at trough, 3 h and 12 h (collecting point 1, 2 and 3) after drug administration, and the other three collecting points were designated in the dialysis process: 0 h, 2 h, 4 h (collecting point 4, 5 and 6), and the dialysis procedure begun at 19 h and finished at 23 h after drug administration. The exposure levels of nirmatrelvir and ritonavir were determined using this method, and the results (Figure 3) showed huge exposure differences between patients (patient 1 compared with other three patients) and patient 3 may suffer from an absorption delay as collecting point 3 (12 h after administration) exhibits a higher exposure level compared with other collecting point. The study reported that within 30 days, nirmatrelvir/ritonavir can significantly decrease the risk of hospital admission or death of a positive outpatient SARS-CoV-2 test [22, 23]. Except for ISDs, many other drugs may have drug-drug interactions with nirmatrelvir/ritonavir, for example, the antiepileptic drugs [24], conazole drugs [25], and so on [26], so the coprescription of nirmatrelvir/ritonavir with other drugs necessitates the close monitoring of their exposure. In renal impairment patients, the exposure of nirmatrelvir increased along with the increasing renal impairment [27], and renal impairment patient may require a dose reduction. In the dialysis patients, nirmatrelvir/ritonavir in this regimen was well tolerated [28]. In our study, the nirmatrelvir/ritonavir exposure levels in plasma could be rapidly eliminated by hemodialysis (collecting points 4, 5, and 6), which was reported by Lingscheid et al. [29]. Nirmatrelvir/ritonavir will not accumulate in the dialysis patients, and dialysis maybe helpful in the rescue of overdose.

## 4. Conclusion

Here, this study presents a novel, sensitive, and fully validated LC-MS/MS method for the simultaneous quantification of nirmatrelvir and ritonavir in human plasma. The linear ranges were established at 2.0–5000 ng/mL for both nirmatrelvir and ritonavir with only 100 *μ*L plasma sample required for analysis. The sample pretreatment was completed by a simple protein precipitation method, with the running time optimized at 3.65 min. Hemodialysis can rapidly remove nirmatrelvir and ritonavir, which may benefit for the overexposure rescue.

## Figures and Tables

**Table 1:** Mass spectrometry parameters of analytes and IS.

Analytes	MRM transition *m*/*z* (*Q*1⟶*Q*3)	Fragmentor (*V*)	CE (eV)
Nirmatrelvir	500.2⟶110.1	125	18
Ritonavir	721.3⟶296.1	140	17
Selinexor (IS)	444.1⟶282.0	120	19

**Table 2:** The regression parameters of calibration curves of analytes.

Analytes	Regression type	Linear range (ng/mL)	Regression equations	*r*
Nirmatrelvir	Linearity	2.0–5000	*Y* = 37.5581 *∗* *x* *+* 8.6239	0.9985
Ritonavir	Linearity	2.0–5000	*Y* = 30.3743 *∗* *x* − 0.0019	0.9956

**Table 3:** Inter- and intraday precision and accuracy of analytes (*n* *=* 5).

Analytes	Nominal concentration (ng/mL)	Interday measured concentration (ng/mL) ± SD	RSD (%)	RE (%)	Intraday measured concentration (ng/mL) ± SD	RSD (%)	RE (%)
Nirmatrelvir	5	4.8 ± 0.4	7.9	−3.9	5.0 ± 2.2	9.9	0.1
	1000	983.3 ± 89.1	9.1	−1.7	982.3 ± 78.0	7.9	−1.8
	2000	2050 ± 105.6	5.2	2.5	2088 ± 104.4	5.0	4.4

Ritonavir	5	4.8 ± 0.7	13.6	4.1	5.1 ± 0.7	13.4	1.5
	1000	1031 ± 118.7	11.5	3.1	1078 ± 18.1	1.7	7.8
	2000	2088 ± 100.4	4.8	4.4	1998 ± 16.2	0.8	−0.1

**Table 4:** Recovery and matrix effect of analytes.

Analytes	Nominal concentration (ng/mL)	Recovery		Matrix effect	
		Mean (%) ± SD	RSD (%)	Mean (%) ± SD	RSD (%)
Nirmatrelvir	5	98.7 ± 6.5	6.6	87.1 ± 3.7	4.2
	1000	92.0 ± 4.4	4.8	97.8 ± 1.6	1.6
	2000	106.7 ± 2.0	1.9	87.8 ± 3.5	3.9

Ritonavir	5	105.8 ± 8.0	7.5	91.7 ± 9.1	9.9
	1000	89.3 ± 7.7	8.6	87.8 ± 5.8	6.6
	2000	85.7 ± 7.0	8.1	111.8 ± 7.3	6.5

**Table 5:** Results of stability in the autosampler of analytes (*n* = 3).

Analytes	Nominal concentration (ng/mL)	0 h			12 h			24 h		
		Mean (%) ± SD	RSD (%)	RE (%)	Mean (%) ± SD	RSD (%)	RE (%)	Mean (%) ± SD	RSD (%)	RE (%)
Nirmatrelvir	5	5.1 ± 0.2	4.5	2.0	4.38 ± 0.21	4.8	−12.5	5.4 ± 0.3	4.9	7.2
	1000	1077 ± 32.4	3.0	7.7	908.4 ± 65.5	7.2	−9.2	982.2 ± 29.9	3.0	−1.8
	2000	2140 ± 78.1	3.7	7.0	2004 ± 15.6	0.8	0.2	2054 ± 71.3	3.5	2.7

Ritonavir	5	4.6 ± 0.2	3.6	−8.0	4.51 ± 0.16	3.4	−9.8	4.5 ± 0.2	3.7	−9.1
	1000	1110 ± 25.9	2.3	11.0	1079 ± 73.1	6.8	7.9	897.5 ± 65.2	7.3	10.3
	2000	2120 ± 33.1	1.6	6.0	1918 ± 12.2	0.6	4.1	1940 ± 46.8	2.4	−3.0

**Table 6:** Results of benchtop stability, three frozen-thaw cycles stability, and long-term stability of analytes (*n* = 3).

Analytes	Nominal concentration (ng/mL)	Room temperature 4 h			Three frozen-thaw cycles			Long-term stability		
		Mean (%) ± SD	RSD (%)	RE (%)	Mean (%) ± SD	RSD (%)	RE (%)	Mean (%) ± SD	RSD (%)	RE (%)
Nirmatrelvir	5	4.6 ± 0.3	6.9	−7.5	4.9 ± 0.6	11.2	−1.2	5.4 ± 0.3	4.8	7.2
	1000	908.4 ± 65.5	7.2	−9.2	983.1 ± 67.3	6.8	−1.7	973.4 ± 26.2	2.7	−2.7
	2000	1851 ± 26.0	1.4	−7.5	2053 ± 110.3	5.4	2.7	2055 ± 31.9	1.6	2.8

Ritonavir	5	4.5 ± 0.2	3.4	−9.8	4.8 ± 0.6	12.4	−5.0	4.5 ± 0.2	3.7	−9.1
	1000	1076 ± 32.0	3.0	7.6	1076 ± 76.6	7.1	7.6	951.7 ± 53.8	5.7	−4.8
	2000	1873 ± 95.5	5.1	−6.4	2101 ± 106.2	5.1	5.0	1806 ± 39.1	3.8	−9.7

## Data Availability

The data used to support the findings of this study are available from the corresponding author upon request.
